# Giant Hyperplastic Polyp at the Gastroesophageal Junction: A Rare Provocateur of Upper Gastrointestinal Bleeding

**DOI:** 10.7759/cureus.43269

**Published:** 2023-08-10

**Authors:** Yumna Shahid, Muhammad Ibrahim Saeed, Amna S Butt

**Affiliations:** 1 Gastroenterology, Aga Khan University Hospital, Karachi, PAK

**Keywords:** hyperplastic polyp of the gastroesophageal junction, medical management, histological features, endoscopic resection, upper gastrointestinal bleeding

## Abstract

Esophageal hyperplastic polyps (HPs) are a rare benign polypoidal growth most commonly resulting from gastroesophageal reflux disease (GERD). The lesion is asymptomatic in most patients unless large enough to cause luminal obstruction or gastrointestinal bleeding. The treatment of choice is endoscopic resection if it becomes symptomatic. Here, we report a case of a 51-year-old woman presenting with dyspeptic symptoms and upper gastrointestinal bleeding. An upper gastrointestinal endoscopy showed a large polyp with active oozing of blood at the gastroesophageal junction (GEJ), which was removed endoscopically after injecting adrenaline at its base. Histopathological analysis was suggestive of HPs.

## Introduction

Hyperplastic polyps (HPs) of the esophagus or gastroesophageal junction (GEJ) are rare lesions with the exact prevalence being unknown. Generally benign esophageal lesions account for <1% of all esophageal tumors, and the majority are asymptomatic slow-growing tumors with a minimal malignant potential. Giant esophageal polyps are extremely rare and only account for 0.03% of cases of all esophageal lesions [1]. The only indication for benign esophageal lesion removal is when they become symptomatic or when a tissue diagnosis is required [2-4].

The most common location of HPs in the upper gastrointestinal tract is the gastric antrum, and these are mostly small in size (1-2 cm) [5]. In the esophagus, the GEJ is the most common site, followed by the distal and mid-esophagus [1,6]. Histologically, HPs are characterized by an inflamed stroma with a variety of inflammatory cells, such as plasma cells, eosinophils, and fibroblasts [7]. Management options include medical therapy with acid suppression agents or endoscopic resection of symptomatic polyps [1]. We report an extremely rare case of a giant HP of 7 cm in length at the GEJ presenting as a cause of gastrointestinal bleeding.

## Case presentation

A 51-year-old female presented to the gastroenterology outpatient clinic with complaints of abdominal pain and exertional shortness of breath for one week. She had two episodes of melena the day before she presented to the clinic. The pain was in the epigastrium region, and it was burning in character, non-radiating, aggravated by meals, and relieved by taking antacids. She also reported occasional intermittent vomiting. On examination, she looked pale and was tachycardic with a pulse rate of 102 beats per minute. The abdomen was soft and non-tender with no visceromegaly. A digital rectal examination showed black-colored stools. She was admitted and managed with intravenous omeprazole, intravenous fluids, and packed red blood cell transfusions. A baseline workup was done, which showed a hemoglobin level of 8.3 gm/dl, a drop from 11.6 g/dl in just two months.

**Figure 1:**
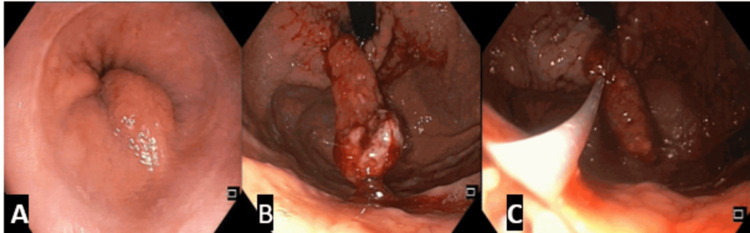
Endoscopic images showing (A) a polypoidal lesion arising from the GEJ, (B) a large pedunculated polyp hanging down in the stomach from the distal esophagus (GEJ) on retroflection, and (C) hot snare polypectomy.

Upper gastrointestinal endoscopy revealed a large polyp with its base located at the GEJ (Fig. 1A). During the procedure, active oozing of blood was noticed (Fig. 1B), which was successfully treated with sclerotherapy followed by hot snare polypectomy in pieces because the polyp was moving up in the esophagus intermittently during the procedure, leading to difficulty in grasping the base of the polyp (Fig. 1C). Argon plasma coagulation was applied at the polypectomy site to minimize the risk of bleeding. The size of the resected polyp was approximately 7 cm.

**Figure 2:**
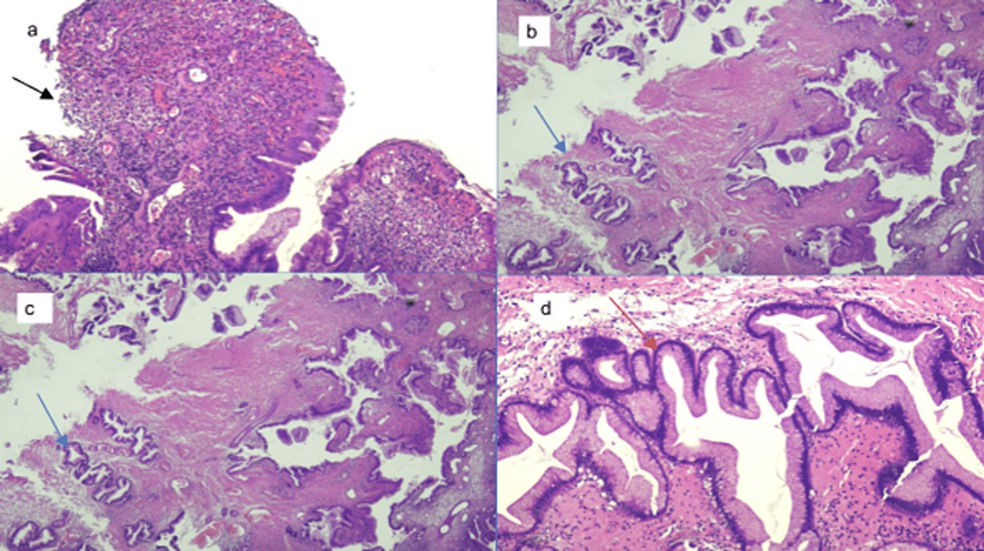
(A) (Hematoxylin and eosin (H&E) 4X magnification) Hyperplastic polyp with surface ulceration and granulation tissue formation (black arrow). (B & C) (H&E 4X magnification) Hyperplastic polyp with variably dilated mucus-secreting glands and intervening fibrotic and mildly inflamed stroma (blue arrows). (D) (H&E 20X magnification) High-power view of mucous-secreting glands. There is no adenomatous change (red arrow).

Histopathological examination showed dense acute and chronic inflammation with surface ulceration and inflamed granulation tissue along with proliferative reactive endothelial cells and fibroblasts (Fig. 2a). The dilated, tortuous glands were lined by mucin-secreting columnar epithelium (Fig. 2b, 2c, 2d). These features were consistent with the histological diagnosis of inflamed HPs.

**Figure 3:**
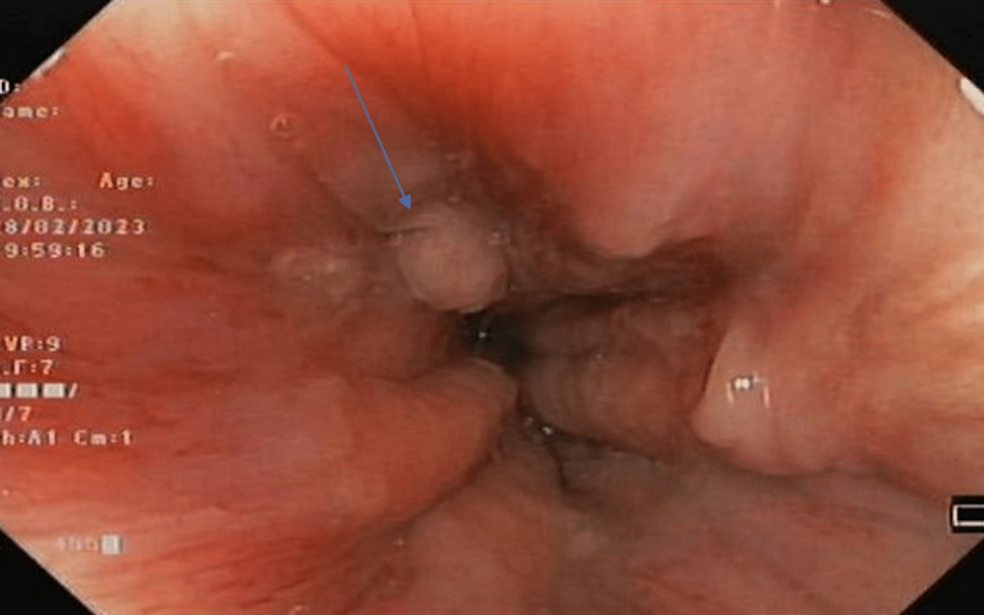
Upper gastrointestinal endoscopy after two years showing a small polyp (arrow) at the same site of the previously removed polyp.

The patient was discharged on proton pump inhibitors and prokinetics. A surveillance endoscopy after two years showed a diminutive polyp at the same location (GEJ) (Fig. 3), which was removed with biopy forcep and sent for histopathology. The histopathology report showed no signs of dysplasia or malignancy. The patient is still under endoscopic surveillance for recurrence of the primary disease or malignant transformation as there is a risk of neoplasia in polyps greater than 1 cm [8].

## Discussion

Although uncommon, a wide variety of pathologies can produce polypoidal lesions in the esophagus or at the GEJ. These include squamous papilloma, leiomyoma, Barrett’s esophagus-associated polypoid dysplasia, polypoid carcinoma, fundic gland polyps, and HPs [4]. HP of the GEJ is an infrequent occurrence, and very limited data are available regarding its etiology, associations, and clinical characteristics. The first study that evaluated its clinical spectrum was performed by Kevin B. Long, and it included 46 cases of GEJ HPs [5]. The study concluded that GEJ HPs are benign and more common in men, predominantly affect younger age groups, and are mostly <1 cm in size (86%), and their recurrence rate is 2% at 20 months [5]. The distinctive feature in our case is an unusually large size (7 cm) polyp at the GEJ, and its recurrence occurred after two years.

Another study conducted on GEJ HPs by Abraham et al. reported a high association with gastroesophageal reflux disease (GERD) and Barrett’s esophagus, i.e., 48% and 15%, respectively [7]. Other possible causes could be medication-induced esophagitis, infection, polypectomy sites, and photodynamic therapy. Hence, the study emphasized the importance of biopsy from non-polypoidal mucosa to determine the underlying cause of HPs [7]. Esophageal and GEJ HPs usually do not undergo a malignant transformation as opposed to gastric and colonic HPs that can be malignant on presentation [9]. Buyukasik et al. conducted a retrospective study on upper gastrointestinal endoscopies and found that the most common location of HPs is the gastric antrum (43%), while the lower esophagus is the least common location (4.6%) [10]. This study signifies the unique location of the polyp in our case.

On the extensive literature review, we found case reports previously published on GEJ polyps. One case report was of an inflammatory fibroid polyp in a five-year-old girl [10], another case was of a sessile polyp at GEJ in a young male post-liver transplant [11], and another reported an esophageal HP located on a Schatzki’s ring [12]. Similarly, GEJ HPS as a consequence of reflux esophagitis and an association with neurofibromatosis type 1 have also been described [11,12]. A giant HP of 4 cm size has been reported in gastric cardia in association with a *Helicobacter pylori* infection [13]. To the best of our knowledge, this is the first case of a giant HP measuring around 7 cm in size at the GEJ.

## Conclusions

Diagnosis of GEJ polyps can be easily missed in asymptomatic cases. Therefore, an upper gastrointestinal endoscopy should be performed in patients presenting with anemia or occult gastrointestinal bleeding to rule out HPs as a cause of upper gastrointestinal bleeding. Following resection of larger polyps, endoscopy surveillance is warranted to assess for the recurrence or development of neoplasia.
